# Cloud Computing Based Systems for Healthcare

**DOI:** 10.1155/2014/692619

**Published:** 2014-04-15

**Authors:** Vladimir Stantchev, Ricardo Colomo-Palacios, Michael Niedermayer

**Affiliations:** ^1^SRH Hochschule Berlin, Ernst-Reuter-Platz 10, 10587 Berlín, Germany; ^2^Østfold University College, B R A Veien 4, 1783 Halden, Norway; ^3^Fraunhofer Institute for Reliability and Microintegration, Gustav-Meyer-Allee 25, 13355 Berlin, Germany

The emergence of cloud computing leads to new developments for diverse application domains. This is particularly true for healthcare with its tremendous importance in today's society, thus making it worth to investigate the relevant perspectives and insights. In this special issue, readers will find the foundations together with cutting-edge developments in the state-of-the-art of cloud computing based systems for healthcare.

Cloud computing is getting increasing attention and represents nowadays one of the most important research topics in computing science and information systems. Cloud computing refers to both the applications delivered as services over the Internet and the hardware and software systems within the data centers which provide those services. Cloud is now seen as a valid strategy and specific applications based on these technologies have become widespread.

Healthcare, as with any other service operation, has been impacted by the cloud computing phenomenon with the literature reporting both benefits and challenges of cloud computing in the area. However, the evolving nature of science and technology creates new scenarios that must be studied using interdisciplinary and holistic means.

The aim of this special issue was to collect innovative and high-quality research contributions regarding the advances in the healthcare domain that are enabled by the use of cloud computing architectures and techniques. The focus is intended to be integral for cloud computing in healthcare, but emphasizing not only the IT side of the phenomenon but also the managerial and the health practitioner side.

Editors received a considerable amount of submissions that were peer-reviewed by top experts in the field. Based on the reviews and our reading of the papers, editors selected seven high-quality ones to be published. Contributions of these papers are summarized as follows.

Two contributions deal with scenarios where cloud computing can serve as an enabler for improved decision making and contribute to systemic improvements in healthcare domain. In “*Usalpharma: a cloud-based architecture to support Quality Assurance training processes in health area using Virtual Worlds*” by F. J. García-Peñalvo et al., the authors discuss ways cloud-based architectures can extend and enhance the functionality of training environments based on Virtual Worlds with focus on training processes in Quality Assurance for pharmaceutical laboratories. In “*Cloud based meta-learning system for predictive modeling of biomedical data*” by M. Vukicevic et al., the authors propose a cloud-based system that integrates a meta-learning framework for ranking and selection of the best predictive algorithms for data at hand and open-source big data technologies for analysis of biomedical data.

Two contributions focus on the topics of risk and security as management issues in cloud computing based systems for healthcare. In “*Proposal for a security management in cloud computing for health care*” K. by Haufe et al., the authors propose a framework that aims to cover the most important security processes related to cloud computing in the healthcare sector. The approach considers both the standards of the ISO 27000 family, as well as specific aspects of healthcare organizations using cloud computing. In “*Risks and crises for healthcare providers: the impact of cloud computing*” by R. Glasberg et al., the multidisciplinary team of authors analyze risks and crises for healthcare providers and discuss the impact of cloud computing in such scenarios.

Three contributions deal with specific healthcare-related use cases of cloud computing in diverse application scenarios. In “*SAMuS: service-oriented architecture for multisensor surveillance in smart homes*,” S. Van Hoecke et al. present the design of a service-oriented architecture (SOA) for multisensor surveillance in smart homes. The solution is evaluated by building a smart Kinect sensor that is able to dynamically switch between IR and RGB and improves person detection by incorporating feedback from pressure sensors within the SOA. In “*A cloud-based X73 ubiquitous mobile healthcare system: design and implementation*” by Z. Ji et al., a ubiquitous mobile healthcare uHealth system is presented. It is based on the ISO/IEEE11073 personal health data (PHD) standards (X73) and cloud computing techniques. In “*An expert fitness diagnosis system based on elastic cloud computing,*” K. C. Tseng et al. describe an expert diagnosis system based on cloud computing that is able to classify a user's fitness level based on supervised machine learning techniques. This system uses parameters such as user's physiological data, age, gender, and body mass index (BMI) and utilizes an elastic algorithm based on Poisson distribution to allocate computation resources dynamically.

The special issue editors would like to take this opportunity to thank the authors for their papers and the reviewers for their valuable comments and suggestions. Special thanks also to the editorial team for its help and also for providing us an opportunity to edit this special issue.

